# Antigen Sensitization Influences Organophosphorus Pesticide–Induced Airway Hyperreactivity

**DOI:** 10.1289/ehp.10694

**Published:** 2008-01-02

**Authors:** Becky J. Proskocil, Donald A. Bruun, Jesse K. Lorton, Kirsten C. Blensly, David B. Jacoby, Pamela J. Lein, Allison D. Fryer

**Affiliations:** 1 Department of Physiology and Pharmacology; 2 Center for Research on Occupational and Environmental Toxicology and; 3 Division of Pulmonary and Critical Care Medicine, Oregon Health & Science University, Portland, Oregon, USA

**Keywords:** airway hyperreactivity, asthma, atopy, eosinophils, organophosphorus pesticides, parathion, sensitization

## Abstract

**Background:**

Recent epidemiologic studies have identified organophosphorus pesticides (OPs) as environmental factors potentially contributing to the increase in asthma prevalence over the last 25 years. In support of this hypothesis, we have demonstrated that environmentally relevant concentrations of OPs induce airway hyperreactivity in guinea pigs.

**Objectives:**

Sensitization to allergen is a significant contributing factor in asthma, and we have shown that sensitization changes virus-induced airway hyperreactivity from an eosinophil-independent mechanism to one mediated by eosinophils. Here, we determine whether sensitization similarly influences OP-induced airway hyperreactivity.

**Methods:**

Nonsensitized and ovalbumin-sensitized guinea pigs were injected subcutaneously with the OP parathion (0.001–1.0 mg/kg). Twenty-four hours later, animals were anesthetized and ventilated, and bronchoconstriction was measured in response to either vagal stimulation or intravenous acetylcholine. Inflammatory cells and acetylcholinesterase activity were assessed in tissues collected immediately after physiologic measurements.

**Results:**

Ovalbumin sensitization decreased the threshold dose for parathion-induced airway hyperreactivity and exacerbated parathion effects on vagally induced bronchoconstriction. Pretreatment with antibody to interleukin (IL)-5 prevented parathion-induced hyperreactivity in sensitized but not in nonsensitized guinea pigs. Parathion did not increase the number of eosinophils in airways or the number of eosinophils associated with airway nerves nor did it alter eosinophil activation as assessed by major basic protein deposition.

**Conclusions:**

Antigen sensitization increases vulnerability to parathion-induced airway hyperreactivity and changes the mechanism to one that is dependent on IL-5. Because sensitization to allergens is characteristic of 50% of the general population and 80% of asthmatics (including children), these findings have significant implications for OP risk assessment, intervention, and treatment strategies.

The prevalence of asthma has been rising over the past 25 years, particularly in children (Mannino et al. 2002). The brevity of this time span strongly suggests that something in the environment is affecting the development of asthma. Coincidentally, organophosphorus pesticides were introduced and have been extensively used during this same time frame (Wessels et al. 2003). Human exposure to organophosphorus pesticides is widespread and occurs both in inner cities, where organophosphorus pesticides are routinely used for control of roaches and rodents (Berkowitz et al. 2003; Landrigan et al. 1999; Lu et al. 2001), and in rural communities, where organophosphorus pesticides are used in agriculture (Fenske et al. 2002; Koch et al. 2002; Schwartz 1999). Humans are exposed via inhalation, absorption through skin or eyes, and ingestion (Gallo and Lawryk 1991). Children are at particularly high risk for exposure (Faustman et al. 2000; Fenske et al. 1990; Gurunathan et al. 1998; Landrigan et al. 1999) and may retain higher concentrations of organophosphorus pesticide metabolites than adults (Barr et al. 2004). Epidemiologic studies report a positive association between organo-phosphorus pesticide exposure and asthma in both children (Eskenazi et al. 1999) and adults (Deschamps et al. 1994; Hoppin et al. 2006, 2007; Senthilselvan et al. 1992).

Organophosphorus pesticides, at environmentally relevant concentrations that do not inhibit acetylcholinesterase (AChE), may precipitate asthma in humans (Bryant 1985) and cause airway hyperreactivity, a key feature of asthma, in guinea pigs (Fryer et al. 2004; Lein and Fryer 2005). Under normal circumstances, airway tone is maintained by parasympathetic nerves that release acetylcholine onto M3 muscarinic receptors, causing airway smooth muscle to contract. Acetylcholine release is limited by inhibitory M2 muscarinic receptors on parasympathetic nerves (Fryer and Maclagan 1984). In animals exposed to low concentrations of organophosphorus pesticides, neuronal M2 muscarinic receptor function is lost, increasing release of acetylcholine and potentiating bronchoconstriction (Fryer et al. 2004; Lein and Fryer 2005).

Asthma and antigen challenge–induced airway hyperreactivity are characterized by airway eosinophilia (Adamko et al. 2005; Wenzel 2003). Antigen sensitization, without challenge, causes eosinophils to cluster around airway nerves in guinea pigs (Adamko et al. 2003), a finding also reported in human asthma (Costello et al. 1997). Antigen inhalation activates eosinophils, causing release of major basic protein that binds to and inhibits neuronal M2 muscarinic receptors leading to airway hyperreactivity (Fryer and Jacoby 1992; Jacoby et al. 1993). Antigen sensitization may also influence airway hyperreactivity triggered by other factors. For example, viral infection, which exacerbates asthma (Johnston et al. 1995; Nicholson et al. 1993), causes airway hyperreactivity by inhibiting neuronal M2 receptor function (Fryer and Jacoby 1991). In nonsensitized animals, virus-induced hyperreactivity does not require the presence of eosinophils around airway nerves (Adamko et al. 1999); however, with sensitization, the mechanism of virus-induced hyperreactivity changes to become eosinophil dependent (Adamko et al. 1999). Here we show that prior sensitization to antigen not only changes the mechanism of organophosphorus pesticide-induced airway hyperreactivity, but also increases vulnerability to organophosphorus pesticides.

## Materials and Methods

### Animals

Specific pathogen-free female Dunkin-Hartley guinea pigs were obtained from Elm Hill Labs (Chelmsford, MA) in filtered crates, housed in a room with high-efficiency particulate-filtered air, and fed a normal diet (Prolab; Agway, Syracuse, NY). We chose guinea pigs for these studies because their lung pharmacology is similar to humans (Canning 2003). In addition, organophosphorus pesticides uniformly cause airway hyper-reactivity in guinea pigs unlike mice, which respond differently depending upon the strain (Smolen et al. 1985, 1986). All guinea pigs were treated humanely with regard for alleviation of suffering in accordance with the standards established by the U.S. Animal Welfare Act as set forth in the National Institutes of Health guidelines (NIH 2002). All protocols involving guinea pigs were approved by the Animal Care and Use Committee at Oregon Health & Science University.

### Ovalbumin sensitization

Guinea pigs (150–200 g) received 3 ip injections of oval-bumin (Sigma-Aldrich Chemical Co., St. Louis, MO) at 6 mg/kg body weight (bw) in sterile phosphate-buffered saline (PBS) on days 1, 3, and 5 (Adamko et al. 1999). Other treatments were not given until 21 days after the last injection of ovalbumin.

### Parathion exposure

Parathion (*o*,*o*-diethyl-*o*-*p*-nitrophenyl phosphorothioate, 99.5% pure) was purchased from Chem Service, Inc. (West Chester, PA). Parathion was suspended in peanut oil, and doses of 0.001–1.0 mg/kg bw or equal volumes of peanut oil (300 μL) were injected sc into the subscapular region (Lein and Fryer 2005). This route of exposure allows for steady release of pesticides, as observed in human exposure (Pope et al. 1991). Peanut oil was administered to controls. After parathion injections, animals were monitored (1, 4, and 24 hr) for signs of cholinergic intoxication (tremors, altered gait, and excessive excretions), which were not observed with any of the doses used in this study. A 10-fold higher concentration of parathion has been used in guinea pigs (Lein and Fryer 2005) without any ill-effects on their health. Physiologic measurements of lung function were performed 24 hr after injection.

### AbIL5

Interleukin (IL)-5 is required for eosinophil maturation, and antibody to IL-5 (AbIL5) was previously shown to deplete eosinophils and prevent airway hyperreactivity in antigen-challenged guinea pigs (Elbon et al. 1995). Monoclonal AbIL5 (TRFK-5) was purchased from BD Pharmingen (San Diego, CA), diluted in sterile PBS to a concentration of 240 μg/kg bw, and administered ip 4 days before physiologic measurements of lung function. An equal volume of PBS was administered to control animals.

### Anesthesia and measurement of pulmonary inflation pressure (Ppi)

We performed physiologic experiments as previously described (Fryer and Jacoby 1992). All guinea pigs were young adults weighing between 340–410 g at the time of testing lung physiology. Guinea pigs were anesthetized with 1.5–1.9 g/kg bw urethane, ip (Sigma-Aldrich Chemical Co.). This concentration of urethane provides anesthesia for 8 hr, and experiments did not extend past 4 hr. Jugular veins were cannulated for drug administration, and one carotid artery was cannulated to monitor blood pressure and heart rate. Both vagus nerves were cut and placed on electrodes submerged in oil for vagal stimulation. Guinea pigs were mechanically ventilated via a tracheal cannula providing a positive pressure and constant volume (1 mL/100 g bw and 100 breaths/min). Animals were given guanethidine (2 mg/kg bw, iv; Sigma-Aldrich Chemical Co.) to deplete noradrenaline and were paralyzed with succinylcholine (10 μg/kg/min, iv; Sigma-Aldrich Chemical Co.) throughout the experiment. Pulmonary inflation pressure was measured on a side arm of the tracheal cannula. Bronchoconstriction was measured as an increase in pulmonary inflation pressure (in mmH_2_O) over the ventilator baseline pressure as previously described (Fryer and Maclagan 1984; Fryer and Wills-Karp 1991; Jacoby and Fryer 1991). Vagus nerves were stimulated at 1-min intervals (2–15 Hz, 10 V, 0.2 msec square waves, 5-sec train duration).

### Measurement of postjunctional M2 and M3 muscarinic receptor function

We administered acetylcholine iv (1–10 μg/kg bw; Acros Organics, Morris Plains, NJ) to test the function of postjunctional M2 muscarinic receptors in cardiac muscle and postjunctional M3 muscarinic receptors on airway smooth muscle. In these experiments, we cut the vagus nerves to eliminate reflex-induced broncho-constriction (Wagner and Jacoby 1999).

### Bronchoalveolar lavage

After physiologic measurements in control and parathion-treated guinea pigs, 50 mL warm PBS containing 10 μg/mL isoproterenol (Sigma-Aldrich Chemical Co.) was flushed through the tracheal cannula and retrieved by syringe in 10-mL aliquots. Cells were washed and resuspended in 10 mL PBS and counted on a hemocytometer (for total cell counts). Cells were also cytospun onto slides and stained with Hemacolor (EMD Chemicals, Inc., Gibbstown, NJ) to obtain differential counts of macrophages, eosinophils, neutrophils, and lymphocytes.

### Histology

Lungs were perfused with PBS via the pulmonary artery, removed, and inflated with 10 mL zinc formalin (Anatech Ltd., Battle Creek, MI). Two transverse sections of the proximal region of two lobes (3–5 mm) were embedded in paraffin and processed for immunohistochemistry. Airway nerves were detected with an Ab specific for PGP 9.5 (Cat. no. 7863-2004; Biogenesis, Poole, England) and eosinophils were stained using chromotrope 2R (C-3143; Sigma-Aldrich Chemical Co.), as previously described (Evans et al. 2001). Light microscopic images were acquired using a video microscope (CoolSnap; PhotoMetrics, Inc., Huntington Beach, CA) and analyzed using Metamorph Imaging System (Universal Imaging Corp., Downingtown, PA). We took consecutive images around four medium-sized airways for each animal. Eosinophils in the airway, including the submucosal region and airway smooth muscle and those associated within 8 μm of nerves (the average diameter of an eosinophil) were counted (per square millimeter airway) and averaged for that animal.

Major basic protein was labeled with a rabbit anti-guinea pig antibody (a generous gift from G.J. Gleich, University of Utah) and detected with Alexa Flour 594 goat anti-rabbit IgG secondary antibody (#A11012; Molecular Probes, Eugene, OR). Consecutive images were taken around four cartilaginous airways for each animal, focusing on smooth muscle and connective tissue immediately surrounding the airway. Slides were analyzed by an independent observer blinded to the experimental conditions. An image with the most intense fluorescence was used to establish the exposure time used for all other images. A 100 × 100 pixel region within the lumen of the airway of each photograph was used to determine background fluorescence. A slide that was not reacted with antibody to major basic protein was used to determine the lower threshold for excluding nonspecific staining. Using Metamorph software (Molecular Devices, Sunnyvale, CA), we adjusted image threshold to measure only extra-cellular major basic protein (to exclude eosinophils). Average fluorescence per area was tabulated for each airway and then averaged for each animal.

### AChE assay

We collected heparinized blood, brain, and PBS-perfused lung to measure AChE activity using the Ellman assay (Ellman et al. 1961), as previously described (Lein and Fryer 2005).

### Statistics

We analyzed a minimum of three to four guinea pigs, but more typically five to seven were analyzed per experimental condition. The effects of sensitization and parathion on baseline body weight, pulmonary inflation pressure, blood pressure, and heart rate were analyzed by one-way analysis of variance (ANOVA) analysis using the Bonferroni correction. We compared bronchoconstriction in response to vagal stimulation and to intravenous acetylcholine, using two-way ANOVA for repeated measures. Changes in AChE activity, leukocytes in bronchoalveolar lavage fluid, and eosinophils in airways were analyzed by one-way ANOVA analysis using the Fisher least significant difference post hoc test. All values are expressed as the mean ± SE.

## Results

### Baseline physiologic parameters

Nonsensitized and ovalbumin-sensitized guinea pigs were age matched. Sensitized animals were not challenged with antigen prior to experimentation. Guinea pig weight (360 ± 11 g in nonsensitized vs. 405 ± 14 g in sensitized animals), baseline pulmonary inflation pressure (100 ± 6 mmH_2_O in nonsensitized vs. 89 ± 3 mmH_2_O in sensitized animals), heart rate (325 ± 11 beats/min in nonsensitized vs. 286 ± 7 beats/min in sensitized animals), and blood pressure (51 ± 3 systolic/25 ± 2 diastolic mmHg in nonsensitized vs. 45 ± 2 systolic/ 23 ± 1 diastolic mmHg in sensitized animals) were not changed by parathion treatment.

### Bronchoconstriction

**Figure 1:**
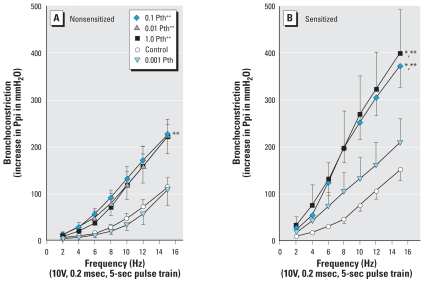
Parathion (Pth) potentiates vagally stimulated bronchoconstriction in nonsensitized (*A*) and sensitized (*B*) guinea pigs. Electrical stimulation of both vagus nerves (2–15 Hz, 10 V, 0.2 msec, 5-sec pulse train) caused frequency-dependent bronchoconstriction as measured by an increase in Ppi. Vagally induced bronchoconstriction was not significantly different in nonsensitized control and sensitized control animals. In nonsensitized animals, 0.01 mg/kg bw, 0.1 mg/kg bw, and 1.0 mg/kg bw parathion sc significantly increased vagally induced bronchoconstriction, whereas 0.001 mg/kg bw parathion sc had no effect. In sensitized animals, 0.1 mg/kg bw and 1.0 mg/kg bw parathion sc significantly increased vagally induced bronchoconstriction; 0.001 mg/kg bw parathion sc also increased vagally induced bronchoconstriction in sensitized guinea pigs, although this change did not reach statistical significance. The increase in vagally induced bronchoconstriction in response to 0.1 mg/kg bw and 1.0 mg/kg bw parathion sc was significantly greater in sensitized animals than in nonsensitized animals. Data are presented as mean ± SE (*n* = 3–13 guinea pigs). *p < 0.05 compared with similar treatment in nonsensitized animals. **p < 0.05 compared with respective control animals.

Electrical stimulation of both vagus nerves caused frequency-dependent bronchoconstriction that was not significantly altered by sensitization to oval-bumin (Figure 1). In nonsensitized animals, doses of parathion ≥ 0.01 mg/kg bw potentiated vagally induced bronchoconstriction (Figure 1). This potentiation was significantly enhanced by prior sensitization to ovalbumin. Additionally, low doses of parathion (0.001 mg/kg bw) that had no effect on vagally induced bronchoconstriction in non-sensitized animals increased vagally induced bronchoconstriction in guinea pigs that had been sensitized to ovalbumin (Figure 1, light blue triangles). Thus, parathion-induced hyperreactivity is exacerbated by sensitization. In addition, sensitization decreases the threshold dose of parathion needed to cause airway hyperreactivity.

**Figure 2:**
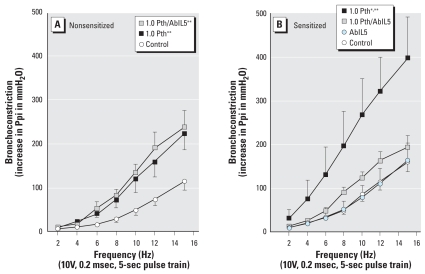
Differential effects of AbIL5 on parathion (Pth) potentiation of vagally -induced bronchoconstriction in nonsensitized (*A*) versus sensitized (*B*) animals. Electrical stimulation of the vagus nerves caused frequency-dependent bronchoconstriction, and responses in control and 1.0 mg/kg bw parathion-treated guinea pigs are graphed as in Figure 1. AbIL5 (240 μg/kg bw, ip) given prior to 1.0 mg/kg bw parathion did not protect parathion-induced airway hyperreactivity in nonsensitized guinea pigs but did in sensitized guinea pigs. AbIL5 alone did not affect airway reactivity. Data are presented as mean ± SE (*n* = 4–13 guinea pigs). *p < 0.05 compared with similar treatment in nonsensitized animals; **p < 0.05 compared with respective control animals.

**Figure 3:**
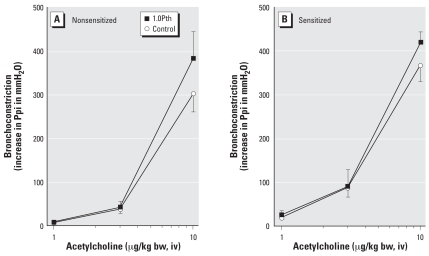
Parathion (Pth) does not change acetylcholine-induced bronchoconstriction in either nonsensitized (*A*) or sensitized (*B*) guinea pigs. Exogenous acetylcholine (1–10 μg/kg bw, iv) induced bronchoconstriction in vagotomized animals, and this response was not affected by either sensitization or parathion (1.0 mg/kg bw, sc). Data are presented as mean ± SE (*n* = 4–13 guinea pigs).

Parathion-induced airway hyperreactivity was prevented by pretreatment with AbIL5 in sensitized but not in nonsensitized guinea pigs (Figure 2). In the absence of parathion, AbIL5 did not have any effect on vagally induced bronchoconstriction (Figure 2, blue circles). Intravenous acetylcholine administered to vagotomized animals caused dose-dependent bronchoconstriction that was not altered by sensitization or by parathion (Figure 3).

### Bradycardia

**Figure 4:**
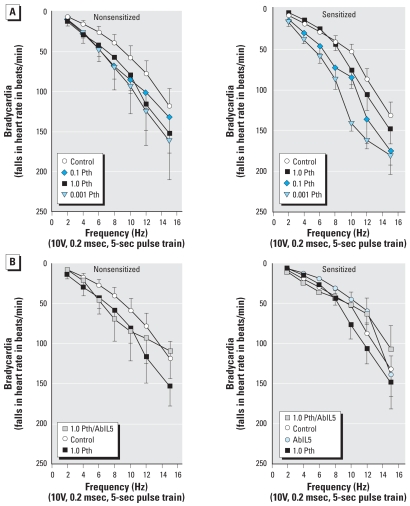
Parathion (Pth) potentiation of vagally induced bradycardia is not changed by sensitization. Electrical stimulation of both vagi (2–15 Hz, 10 V, 0.2 msec, 5-sec pulse train) caused frequency-dependent bradycardia. (*A*) Parathion slightly but not significantly increased vagally induced bradycardia in both non-sensitized and sensitized animals. In contrast to the lung, however, sensitization did not exacerbate parathion effects. (*B*) AbIL5 did not affect vagally induced bradycardia in either nonsensitized or sensitized animals. Data are presented as mean ± SE (*n* = 3–12 guinea pigs).

**Figure 5:**
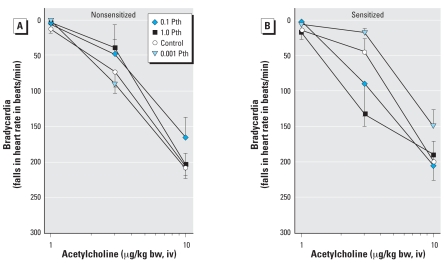
Acetylcholine-induced bradycardia does not differ with either parathion (Pth) exposure or sensitization. Bradycardia induced by increasing doses of exogenous acetylcholine (1–10 μg/kg bw, iv) was measured in nonsensitized (*A*) and sensitized (*B*) guinea pigs exposed to parathion or vehicle. All animals were vagotomized prior to administration of acetylcholine. Data presented as mean ± SE (*n* = 3–13 guinea pigs).

Vagal stimulation caused frequency-dependent bradycardia that was not affected by sensitization (Figure 4A). Parathion slightly, but not significantly, potentiated vagally induced bradycardia in nonsensitized guinea pigs, and this response was not altered by sensitization (Figure 4A) or by pretreatment with AbIL5 (Figure 4B). Neither sensitization nor parathion affected acetylcholine-induced bradycardia (Figure 5).

### AChE activity

**Figure 6:**
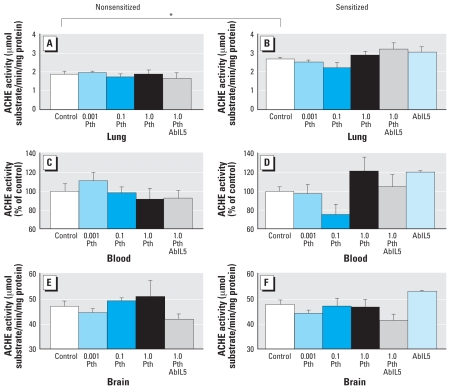
Doses of parathion (Pth; mg/kg bw) that cause hyperreactivity do not inhibit AChE. Neither parathion nor sensitization inhibited AChE activity in the lung (*A*, nonsensitized; *B*, sensitized), blood (*C*, nonsensitized; *D*, sensitized), or brain (*E*, nonsensitized; *F*, sensitized) of guinea pigs. However, in lung, sensitization increased AChE activity. Data are presented as mean ± SE (*n* = 3–17 guinea pigs). *p < 0.005.

Sensitization alone significantly increased AChE activity in lung; however, at the doses tested in this study, parathion did not inhibit AChE activity in brain, lung, or blood in either nonsensitized or sensitized guinea pigs (Figure 6).

### Bronchoalveolar lavage

**Figure 7:**
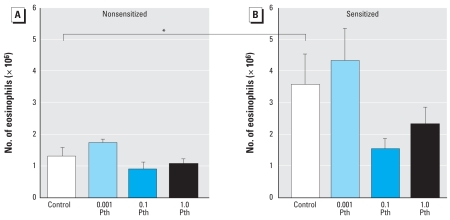
Eosinophils from bronchoalveolar lavage are affected by sensitization and parathion (Pth; mg/kg bw). (*A*) In nonsensitized guinea pigs, eosinophils in the bronchoalveolar lavage were not affected by parathion. (*B*) Sensitization significantly increased eosinophils, and 0.1 mg/kg bw and 1.0 mg/kg bw parathion reduced eosinophils in bronchoalveolar lavage, although this effect was not statistically significant. AbIL5 inhibited eosinophils recovered in the bronchoalveolar lavage of sensitized guinea pigs (3.7 ± 0.95 × 10^6^ eosinophils versus 1.8 ± 0.55 × 10^6^ eosinophils in animals treated with AbIL5). Data presented as mean ± SE (*n* = 3–17 guinea pigs). *p < 0.05.

Total inflammatory cells in bronchoalveolar lavage fluid were significantly increased by sensitization (9 ± 1.3 million cells in nonsensitized versus 13 ± 1.2 million cells in sensitized animals, *p* < 0.05). Neither parathion (0.001–1 mg/kg bw, sc) nor AbIL5 altered total cells recovered from either nonsensitized or sensitized animals (data not shown). A comparative analysis of macrophages, neutrophils, lymphocytes, and eosinophils indicates that the increase in total cells observed in sensitized guinea pigs primarily reflects an increase in eosinophils (data not shown). Treatment with AbIL5 decreased eosinophils in sensitized guinea pigs (3.7 ± 0.95 × 10^6^ eosinophils vs. 1.8 ± 0.55 × 10^6^ eosinophils in animals treated with AbIL5). In sensitized animals, the two higher doses of parathion decreased eosinophils in the lavage, although this was not statistically significant (Figure 7). Other inflammatory cells were not affected by parathion in sensitized guinea pigs (data not shown). In nonsensitized guinea pigs, parathion had no effect on eosinophils (Figure 7) or other inflammatory cell populations (data not shown).

### Airway histology

**Figure 8:**
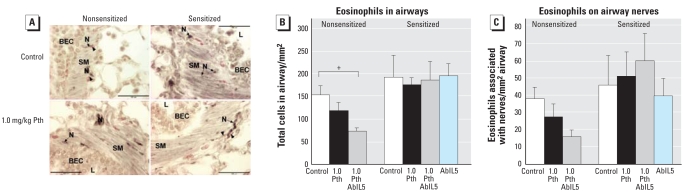
Quantification of eosinophils in lungs and along airway nerves. Abbreviations: BEC, bronchial epithelial cells; L, lumen; N, nerve; Pth, parathion; SM, smooth muscle. (*A*) Representative images of airways from nonsensitized and sensitized, control, and 1.0-mg/kg bw parathion-treated guinea pigs (scale bar = 100 μm). Airway nerves (N) were immunolabeled for PGP 9.5 (purple–black staining) and eosinophils were stained red with chromotrope 2R. Eosinophils associated with airway nerves are indicated by an arrowhead (*A*). In nonsensitized guinea pigs, parathion decreased eosinophils in peribronchial regions (*B*) and around airway nerves (*C*). Sensitization did not change the number of eosinophils in the airway (*B*) nor did parathion have an effect on the number of eosinophils in airways (B) or along airway nerves (*C*) in sensitized guinea pigs. ^+^*p* < 0.05 compared with respective control animals (*n* = 4–7 guinea pigs).

Because parathion decreased eosinophils in the lavage of sensitized animals, we examined airway histology to determine whether eosinophils remained in lung tissue. In sensitized guinea pigs, neither parathion nor AbIL5 affected eosinophils in airway tissues or eosinophils associated with nerves (Figure 8). In nonsensitized guinea pigs, parathion decreased eosinophils in airway tissues as well as in eosinophils specifically associated with airway nerves (Figure 8).

### Eosinophil degranulation

**Figure 9:**
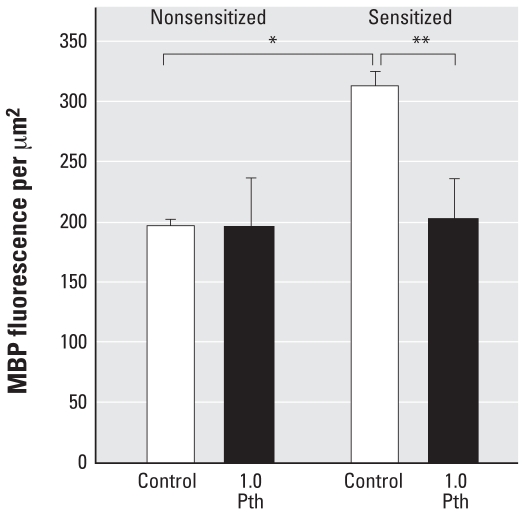
Quantification of eosinophil major basic protein in lung tissue. Extracellular major basic protein was quantified by fluorescent immunohistochemistry in peribronchial regions. In nonsensitized guinea pigs, parathion (Pth; 1.0 mg/kg bw sc) did not cause an increase in major basic protein deposition. Sensitization significantly increased major basic protein deposition, an effect that was attenuated by 1.0 mg/kg bw parathion. Data presented as mean ± SE (*n* = 3–5 guinea pigs). *p < 0.05 compared with similar treatment in nonsensitized animals. **p < 0.05 compared with respective control.

Because parathion decreased eosinophils in the lavage of sensitized animals but did not similarly affect eosinophils in airway tissues, extracellular major basic protein deposition was quantified as an indicator of eosinophil degranulation. Sensitization significantly increased major basic protein deposition in the lungs. Although parathion had no effect on major basic protein deposition in nonsensitized animals, it significantly decreased major basic protein deposition in sensitized animals (Figure 9).

## Discussion

The data presented here show that in adult female guinea pigs, sensitization to allergen increases vulnerability to organophosphorus pesticide-induced airway hyperreactivity. The organophosphorus pesticide parathion potentiated vagally induced bronchoconstriction in nonsensitized guinea pigs as previously shown (Fryer et al. 2004; Lein and Fryer 2005). Sensitization to ovalbumin decreased the threshold dose of parathion required to cause airway hyperreactivity and exacerbated parathion potentiation of vagally induced bronchoconstriction. In addition, administration of AbIL5 before parathion blocked parathion-induced airway hyperreactivity in sensitized but not in nonsensitized guinea pigs. Thus, sensitization increases the ability of organophosphorus pesticides to cause airway hyperreactivity and also changes the mechanism from IL-5 independent to IL-5 dependent.

It has been proposed that organophosphorus pesticide–induced asthma is mediated by inhibition of AChE (Senthilselvan et al. 1992), the enzyme that degrades acetylcholine. Antigen sensitization alone increased AChE activity in lungs. This does not agree with other reports that sensitization decreases AChE activity in tracheal smooth muscle (Mitchell et al. 1991, 1992). We have shown that sensitization of guinea pigs increases eosinophils in the lungs. Inflammatory cells (Kawashima and Fujii 2000), including eosinophils (Lepore et al. 1984), express AChE activity, and there is evidence that eosinophils can induce AChE RNA expression and activity in a neuroblastoma cell line (Durcan et al. 2006). Although there was an increase in AChE activity in the whole lungs of sensitized guinea pigs, this did not affect airway physiology, as both nonsensitized and sensitized guinea pigs had similar increases in bronchoconstriction in response to vagal stimulation (Figure 1). Parathion did not inhibit AChE activity in either nonsensitized or sensitized guinea pigs. Moreover, bronchoconstriction in response to exogenous acetylcholine was not increased by parathion treatment. Together, these data demonstrate that the increased response to vagal stimulation in parathion-treated animals was due to increased acetylcholine release, and not to inhibited AChE activity or increased sensitivity of smooth muscle. Thus, parathion selectively targets nerve function to potentiate vagally induced bronchoconstriction.

Although some eosinophils reside in normal guinea pig lungs, sensitization without antigen challenge is sufficient to recruit additional eosinophils to the lungs (Adamko et al. 1999). Eosinophil presence alone, however, is not sufficient to cause airway hyperreactivity (Adamko et al. 1999, 2003); they must be activated by subsequent antigen challenge (Costello et al. 1997, 2000; Kingham et al. 2003) or viral infection (Adamko et al. 1999, 2003). Thus, it is not the presence of eosinophils in lung but the presence of activated eosinophils around airway nerves that correlates with airway hyperreactivity (Costello et al. 1997, 2000; Kingham et al. 2003).

Organophosphorus pesticides cause a loss of parasympathetic prejunctional M2 muscarinic receptor function (Fryer et al. 2004; Lein and Fryer 2005). Loss of neuronal M2 muscarinic receptor function in antigen-challenged guinea pigs results from binding of major basic protein, an endogenous M2 muscarinic receptor antagonist released from activated eosinophils (Evans et al. 1997; Jacoby et al. 1993). Therefore, it was surprising to find that parathion did not increase eosinophil recruitment to the lungs or to the nerves. Decreased eosinophils may result from activation and degranulation as measured by major basic protein deposition (Verbout et al. 2007). Although low concentrations of organophosphorus pesticides can degranulate mast cells and basophils (Rodgers and Ellefson 1992; Rodgers and Xiong 1997; Xiong and Rodgers 1997), parathion did not increase eosinophil activation in the lungs as measured by deposition of major basic protein.

Sensitization changes the mechanism by which parathion causes airway hyperreactivity to become IL-5 dependent. The ability of sensitization to switch the mechanisms of inflammation to be IL-5 dependent has been reported in mice. Viral infection of mice normally increases interferon but not IL-5, whereas viral infection of sensitized mice increases IL-5 but not interferon (Coyle et al. 1995). Similar results have been found in guinea pigs that have been sensitized prior to viral infection (Adamko et al. 2003). Although IL-5 is a key regulator for eosinophil recruitment and activation (Lopez et al. 1988; Yamaguchi et al. 1988), IL-5 receptors are present on other inflammatory cells (Dewachi et al. 2006; Pierrot et al. 2001; Suttmann et al. 2003). Although we have yet to establish a clear role for eosinophils or other inflammatory cells in parathion-induced hyperreactivity, our data show that sensitization changes the mechanism of parathion-induced hyperreactivity from IL-5 independent to IL-5 dependent.

One possible mechanism underlying potentiation of airway hyperreactivity by parathion in sensitized animals may involve paraoxonase, the enzyme that degrades the active metabolite of parathion. Inflammation decreases paraoxonase activity (Cabana et al. 1996; Van Lenten et al. 1995), which would be expected to increase the biological half-life of the active parathion metabolite. Whether the decrease of paraoxonase activity by inflammation requires eosinophils is not known, but if this were the case, it would explain the requirement for IL-5 in parathion-induced hyperreactivity in sensitized animals. The mechanism by which parathion causes airway hyperreactivity in nonsensitized guinea pigs is currently under investigation. Organo-phosphorus pesticides down-regulate or competitively bind to muscarinic receptors in brain [as reviewed by Jett and Lein (2007)], and by analogy, organophosphorus pesticides may interact directly with neuronal M2 receptors to block their function and increase vagally induced acetylcholine release from parasympathetic nerves, thus potentiating vagally induced bronchoconstriction.

As in the lungs, all doses of parathion slightly shifted vagally induced bradycardia to the right, although the effect was not dose related or significant. This minor shift has been reported before (Lein and Fryer 2005) and occurred regardless of sensitization status or AbIL5 treatment. This parathion-mediated shift in vagally induced bradycardia may be due to selective loss of neuronal M2 muscarinic receptor function on parasympathetic nerves supplying the heart. Because acetylcholine-induced bradycardia, which is mediated by postjunctional M2 receptors, was not altered by parathion, this suggests that prejunctional and postjunctional M2 muscarinic receptors are regulated independently in different tissues. A similar susceptibility of neuronal M2 muscarinic receptors has been reported in guinea pigs treated with double-stranded RNA (Bowerfind et al. 2002). Whether susceptibility of neuronal receptors is mediated by lack of spare receptors or differences in local cellular environments is not known.

In humans, airway hyperreactivity may be subtle and not noticed until measured in a laboratory setting (Empey et al. 1976). In contrast to our guinea pig model, human exposure to allergens and to pesticides is diverse and airway response to these environmental factors can be influenced by genetic background, environmental history, and age of exposure. For example, atopic women not born on farms but who move to a farm and are exposed to pesticides are more likely to develop atopic asthma than atopic women born on farms and exposed to pesticides (Hoppin et al. 2007). The Hoppin et al. study demonstrates that both atopic status and pesticide exposure in humans can impact the development of asthma.

The results of this study confirm and extend previous studies identifying organo-phosphorus pesticides as environmental factors that contribute to asthma by demonstrating that sensitization changes the mechanism underlying organophosphorus pesticide-induced hyperreactivity and increases vulnerability to organophosphorus pesticides. When considered in light of data that estimate over 50% of the U.S. population is sensitized to allergen (Arbes et al. 2005), our findings suggest that allergen sensitization is a major susceptibility factor for asthma that interacts synergistically with organophosphorus pesticides. Recent studies of humans (Almqvist et al. 2008), mice (Card et al. 2007; Carey et al. 2007; Dimitropoulou et al. 2005; Riffo-Vasquez et al. 2007), and guinea pigs (Regal et al. 2006) suggest that sex hormones modify airway responsiveness to various stimuli. Whether sex hormones also influence organophosphorus pesticide-induced airway hyperreactivity remains to be determined. Nonetheless our current work has implications for assessing the risks associated with exposure to organophosphorus pesticides in terms of identifying sensitive subpopulations and setting exposure limits. It also underscores the need for further studies in order to better understand the mechanisms mediating organophosphorus pesticide-induced airway hyperreactivity in allergic versus nonallergic individuals to determine the need for developing customized prevention and therapeutic strategies based on sensitization status.
